# Comparison of the severity of cytopenias with etiologic factors in patients with pancytopenia and bicytopenia

**DOI:** 10.11604/pamj.2019.34.149.18749

**Published:** 2019-11-14

**Authors:** Hakan Sarbay

**Affiliations:** 1 Istanbul Yeni Yüzyil University Faculty of Medicine, Diyarbakir Children Hospital, Pediatric Hematology and Oncology, Istanbul, Turkey

**Keywords:** Pancytopenia, bicytopenia, children

## Abstract

**Introduction:**

The aim of this study was to evaluate the severity of hematological findings according to etiology in patients with pancytopenia and bicytopenia.

**Methods:**

Patients with bicytopenia and pancytopenia who were examined in Diyarbakir Children Hospital Pediatric Hematology and Oncology clinic between June 2017-June 2018 were evaluated retrospectively.

**Results:**

Of the 130 patients included in the study, 73 (56.2%) were male and 43 (43.8%) were female. The mean age of the patients was 4.9 ± 4.86. Forty-five (34.6%) patients had pancytopenia and 85 (65.3%) had bicytopenia. The youngest patient was 1-month old and the oldest patient was 18-year-old. The mean blood count parameters were white blood cell (WBC): 10.207 ± 39.781, neutrophil: 1515 ± 1418, hgb: 9.3 ± 2.3, mean corpuscular volume (MCV): 80 ± 13.1, platelet: 118.823 ± 93.645. Three out of 130 patients had hyperleukocytosis (WBC > 50.000/mm^3^). Vitamin B12 deficiency was detected in 35 patients. When patients with primary hematological disease were compared with patients with secondary causes of cytopenias, a significant difference was found in terms of leukocyte count, hemoglobin level, MCV elevation, and low platelet count.

**Conclusion:**

The determination of the severity of cytopenias in differential diagnosis may be useful in distinguishing primary hematological diseases from secondary causes of pancytopenia and bicytopenia. However, vitamin B12 deficiency in developing countries is one of the most important causes of public health as well as in the etiology of pancytopenia and bicytopenia.

## Introduction

Pancytopenia or bicytopenia is a condition characterized by the decrease of cellular elements in two or three series which develop in childhood due to different etiological reasons. A wide range of causes can be detected in the etiology ranging from transient suppression to malignancies, nutritional deficiencies to inflammatory diseases [1]. When pathophysiologically examined, cytopenias may be due to ineffective hematopoiesis, maturayon pause, infiltration of bone marrow, peripheral sequestration and destruction according to etiologic cause [2]. The severity of clinical signs and symptoms may vary depending on the severity of cytopenia. Neutropenia is defined as mild neutropenia (1000-1500/mm^3^), moderate neutropenia (500-1000/mm^3^) and severe neutropenia (<500/mm^3^) according to absolute neutrophil count. Particularly severe neutropenia carries a risk of serious and life-threatening infections [3]. Thrombocytopenia is classified as thrombocytopenia (100.000-150.000/mm^3^), moderate thrombocytopenia (50.000-100.000/mm^3^) and severe thrombocytopenia (<50.000/mm^3^) according to platelet count. Spontaneous bleeding risk increases significantly when platelet count is less than 20.000/mm^3^ [4]. The aim of this study was to evaluate the severity of hematological findings in patients with pancytopenia and bicytopenia and to provide clinical-laboratory clues and to facilitate the initial diagnosis.

## Methods

Patients with bicytopenia and pancytopenia whom were examined in Diyarbakir Children Hospital Pediatric Hematology and Oncology Clinic between June 2017-June 2018 were evaluated retrospectively. Diagnosis of primary disease, gender, age, white blood cell count, absolute neutrophil count, hemoglobin, platelet count, mean erythrocyte volüme (MCV), vitamin B12 and folate values, increase in liver function tests, history of drug use, hepatosplenomegaly, presence or absence of fever were recorded. Cytopenias were defined as: leukocyte count < 4000/mm^3^, neutrophil count < 1500/mm^3^, platelet count < 150.000/mm^3^, hemoglobin level below -2 standard deviation. The incidence of the diagnoses was calculated. Vitamin B12 deficiency (< 200 ng/ml) determined the vitamin levels of patients identified. Patients were divided into two groups as primary hematological diseases and secondary causes. Groups were compared in terms of gender, age, white blood cell, absolute neutrophil count, hemoglobin, platelet count, MCV, increased liver function tests, hyperleukocytosis, severe cytopenia, hepatosplenomegaly, fever. The patients whose data were not fully reached and who had no definite diagnosis were excluded from the study. The study was approved by the local ethics committee.

**Statistical analysis:** the normality of distribution of continuous variables was tested by ShaphiroWilk test. Mann Whitney U test was used to compare 2 independent group for non-normal data. Chi-square test applied to investigate relationship between 2 categorical variables. Statistical analysis was performed with SPSS for Windows version 24.0 and a p value < 0.05 was accepted as statistically significant.

## Results

Of the 130 patients enrolled in the study, 73 (56.2%) were male and 43 (43.8%) were female. The mean age of the patients was 4.9 ± 4.86. Forty-five (34.6%) patients had pancytopenia and 85 (65.3%) had bicytopenia. The youngest patient was 1 month old, the oldest patient was 18 years old. The mean blood count parameters were WBC: 10.207 ± 39.781, neutrophil: 1515 ± 1418, hgb: 9.3 ± 2.3, MCV: 80 ± 13.1, platelet (plt): 118.823 ± 93.645. Three of 130 patients had hyperleukocytosis (WBC > 50.000/mm^3^). Vitamin B12 deficiency was detected in 35 patients. The mean vitamin B12 level was 113 ± 29. When the etiology was examined, the most common infections and nutritional causes were determined (Table 1). Four (3.2%) patients had a history of chronic drug use. Cytopenia was not thought to be directly related to drug use, as additional diagnoses were present in these patients. Eleven (8.5%) patients had at least a 2-fold increase in liver function tests. In physical examination findings, 29 (22.3%) of the patients had hepatosplenomegaly, 8 (6.2%) had isolated splenomegaly, and 57 (43.8%) had fever. There were significant differences in leukocyte count, hemoglobin level, MCV elevation, and low platelet count in patients with primary hematological disease (group-1) and cytopenias due to secondary causes (group-2). The incidence of fever, organomegaly, hyperleukocytosis and severe cytopenias were high in Group-1 (Table 2).

**Table 1:** Frequency of etiological causes

	n	%
Infections*	63	48.46
Vitamin B12 deficiency	37	21.76
Iron deficiency	17	10.00
EBV	12	7.06
Folat deficiency	5	2.94
B cell ALL	9	5.29
Fanconi aplastic anemia	4	2.35
Metabolic Disease	4	2.35
T cell ALL	2	1.18
SLE	2	1.18
Hemophagocytic lymphohistiocytosis	2	1.18
Hepatitis B	2	1.18
AML	2	1.18
Down syndrome	1	0.59
Autoimmune hemolytic anemia	1	0.59
Niemann-Pick disease	1	0.59
Infant leukemia	1	0.59
Gaucher disease	1	0.59
Congenital nephrotic syndrome	1	0.59
Celiac disease	1	0.59
Hypothyroidism	1	0.59
Hypersplenism	1	0.59

* Respiratory tract infections, gastroenteritis, Brucella, 6. Disease, measles, osteomyelitis

**Table 2:** Comparison of two groups

		Group 1(n=20)	Group 2 (n=110)	P
Gender	Male	5 (25 )	68 (61.8 )	0.002*
	Female	15 (75 )	42 (38.2 )	
Liver function tests	Increase	2 (10 )	9 (8.2 )	0.792
	Normal	18 (90 )	101 (91.8 )	
Drugs	Yes	0 (0 )	4 (3.6 )	0.388
	No	20 (100 )	106 (96.4 )	
Organomegaly	HSM	15 (75 )	14 (12.7 )	0.001*
	Splenomegaly	0 (0 )	8 (7.3 )	
	No	5 (25 )	88 (80 )	
Fever	Yes	15 (75 )	42 (38.2 )	0.002*
	No	5 (25 )	68 (61.8 )	
Leucocytosis (/mm^3^)	>50.000	3 (15)	0 (0)	0.001*
	<50.000	17 (85)	110 (100)	
Neutrophil (/mm^3^)	<500	7 (35)	15 (13.6)	0.019*
	>500	13 (65)	95 (86.4)	
Hemoglobin (gr/dl)	<6	6 (30)	1 (0.9)	0.001*
	>6	14 (70)	109 (99.1)	
Platelet (/mm^3^)	<20.000	3 (15)	1 (0.9)	0.001*
	>20.000	17 (85)	109 (99.1)	

## Discussion

Pancytopenia and bicytopenia is a life-threatening condition in terms of both the underlying cause and the consequences of childhood. Although there may be transient cytopenias due to infections in the etiology, serious diseases related to bone marrow can also be seen. Determining the cause of cytopenia is particularly important for the patient’s follow-up and treatment. When the literature is examined, differences in age and geographic regions are observed in etiology of pancytopenia and bicytopenia patients [5]. In a study of 134 patients in Zimbabwe, the most common cause was megaloblastic anemia, followed by aplastic anemia and acute leukemia [6]. In France, Imbert *et al*. [7] reported the most common cause of myeloid leukemias (42%) in 213 adult patients. In a study involving adult and pediatric patients in Nepal, the most common cause was hypoplastic bone marrow in children (38.1%) and megaloblastic anemia in adults (30.2%) [8]. The most common cause of megaloblastic anemia was found in one of the studies in Pakistan [9]. In my study, infections were the most common cause of pancytopenia and bicytopenia. Cytopenias secondary to infection were followed by vitamin B12 deficiency and acute leukemias. I think vitamin B12 deficiency as a common cause is related to the socioeconomic status of the region where I work. Vitamin B12 deficiency is frequently seen in developing populations. It is one of the most common nutritional deficiencies. In the etiology, inadequate nutrition and chronic bowel diseases are among the most important causes [10]. Naseem *et al.* [11], 571 (57.7%) of 990 children who were referred for bone marrow assessment had pancytopenia or bicytopenia. Aplastic anemia (33.8%), acute leukemia (26.6%) and megaloblastic anemia (13.7%) were the most common etiological factors. The most common symptoms were fever (65.5%), pallor (59%) and hepatomegaly (51.8%). In my study, 57 patients (43.8%) had fever, 29 (22.3%) had hepatosplenomegaly and 8 (6.2%) had isolated splenomegaly. In a study conducted in the capital of our country, 28 patients with bicytopenia were evaluated in 1606 patients. The most common causes were infections with 64.2%, idiopathic thrombocytopenic purpura with 7.1%.

Megaloblastic anemia was detected in only 3.5% of the patients [12]. Unlike my study, I associated this difference in the frequency of B12 deficiency with the development of capital Ankara. Bhatnagar *et al.* [13] reported that 109 children with pancytopenia were diagnosed with megaloblastic anemia and acute leukemias, followed by infections in 3^rd^ place. In a study performed in 105 pancytopenic children, infections were the third most common cause. The most common cause of infection was Kala-Azar [14]. In my study, the most common cause of infection was Epstein-Barr Virus (EBV) infection. In contrast to the study of Gupta *et al.* [14], Kala-Azar was not detected in any patient. Other infectious causes other than EBV were brucellosis, nonspecific upper and lower respiratory tract infections, and gastroenteritis. The reason for not detecting Kala-Azar infection was considered as the primary concern of the department of infectious diseases and the preventions taken by the ministry of health. EBV infections are usually characterized by fever, exudative pharyngitis, lymphadenopathy and hepatosplenomegaly. These findings are especially important in the differential diagnosis with hematological malignancies. Thrombocytopenia, hemolytic anemia, agranulocytosis and hemophagocytic lymphohistiocytosis are among the hematologic complications seen in EBV infections [15, 16]. In a study examining hematological findings related to different viral agents, 21 children with EBV infection had leukopenia 4.8%, leukocytosis 33.3%, neutropenia 14,3%, anemia 23,8%, thrombocytopenia 19% [17]. Cytopenias can develop especially in acute phase during viral infections due to cytokine release, drug side effects, decreased production because of bone marrow suppression, or hemolysis. The biggest difference of cytopenias due to viral infections to hematological malignancies and bone marrow pathologies is that the degree of cytopenia is milder [18]. EBV infection was detected in 12 (7%) patients in my study. It was observed that the degree of cytopenia of these patients was milder than group 1. Acute leukemias and bone marrow disorders are important causes of pancytopenia-bicytopenia in children. Acute leukemias may be presented with leukocytosis and may also be associated with pancytopenia and bicytopenia. The incidence of severe cytopenia which requires transfusion support in bone marrow pathologies is high [19]. In one study, it was found that macrocytosis was more common in bone marrow-derived pathologies and therefore an important finding [20]. In my study, a significant difference was found between the patients who were thought to have primary hematological disease and the patients who were related to the secondary causes of cytopenias.

## Conclusion

In conclusion, determination of the severity of cytopenias may be useful in differentiating primary hematological diseases from cytopenias due to secondary causes and without further examinations in the differential diagnosis. In addition to this, vitamin B12 deficiency should be the first to be considered in the etiology of pancytopenia and bicytopenia especially in developing countries. Vitamin B12 deficiency is so common in public health.

### What is known about this topic

- Pancytopenia and bicytopenia is a life-threatening condition in terms of both the underlying cause and the consequences of childhood;
- A wide range of causes can be detected in the etiology ranging from transient suppression to malignancies, nutritional deficiencies to inflammatory diseases;
- Differences in age and geographic regions are observed in etiology of pancytopenia and bicytopenia patients.

### What this study adds

- Severity of cytopenias may be useful in differentiating primary hematological diseases from cytopenias due to secondary causes and without further examinations;
- Vitamin B12 deficiency should be the first to be considered in the etiology of pancytopenia and bicytopenia in developing countries.

## Competing interests

The author declares no competing interests.
